# Socioeconomic Factors and Caries in People between 19 and 60 Years of Age: An Update of a Systematic Review and Meta-Analysis of Observational Studies

**DOI:** 10.3390/ijerph15081775

**Published:** 2018-08-18

**Authors:** Simone M. Costa, Carolina C. Martins, Mânia Q. C. Pinto, Mara Vasconcelos, Mauro H. N. G. Abreu

**Affiliations:** 1 Department of Dentistry, Universidade Estadual de Montes Claros, Montes Claros, Minas Gerais 39401-089, Brazil; smelocosta@gmail.com (S.M.C.); maniaquadros@gmail.com (M.Q.C.P.); 2 Department of Community and Preventive Dentistry, School of Dentistry, Universidade Federal de Minas Gerais, Belo Horizonte, Minas Gerais 31270-901, Brazil; carolcm10@hotmail.com (C.C.M.); maravas@uol.com.br (M.V.)

**Keywords:** adults, caries, socioeconomic status, epidemiology, meta-analysis

## Abstract

This study is aimed to perform an update of a systematic review and meta-regression to evaluate the effect modification of the socioeconomic indicators on caries in adults. We included studies that associated social determinants with caries, with no restriction of year and language. The Newcastle-Ottawa Scale was used to evaluate the risk of bias. With regard to the meta-analysis, statistical heterogeneity was evaluated by I^2^, and the random effect model was used when it was high. A subgroup analysis was conducted for socioeconomic indicators, and a meta-regression was performed. Publication bias was assessed through Egger’s test. Sixty-one studies were included in the systematic review and 25 were included in the meta-analysis. All of the studies were published between 1975 and 2016. The most frequent socioeconomic indicators were schooling, income, and socioeconomic status (SES). In the quantitative analysis, the DMFT (decayed, missing, filled teeth) variation was attributed to the studies’ heterogeneity. The increase of 10.35 units in the proportion of people with lower SES was associated with an increase of one unit in DMFT, *p* = 0.050. The findings provide evidence that populations with the highest proportions of people with low SES are associated with a greater severity of caries. The results suggest the need for actions to reduce the inequalities in oral health (PROSPERO [CRD42017074434]).

## 1. Introduction

Dental caries affect a high number of adults around the world, and despite the possible applied prevention measures [1,2], a high prevalence of untreated caries was reported between 1990 (34.3%) and 2015 (34.1%), affecting 2.5 billion people worldwide in 2015 [3]. Incorporating social determinants, such as health equity and well-being, into theoretical models help to explain caries inequality among populations [4,5].

In 2016, the World Dental Federation (FDI) approved a new definition of oral health and recognized its multifaceted nature [6]. From this perspective, Healthy People 2020 addressed oral health goals by monitoring and reducing health inequalities [7]. Oral health is an essential component in achieving good general health and it is a fundamental human right to establish a good quality of life and well-being [2]. Previous systematic reviews on socioeconomic indicators and caries in adults showed a relationship between socioeconomic disadvantages and caries. In a qualitative synthesis of 41 studies, schooling, income, and occupation were associated with greater severity of dental caries. However, quantitative data analysis was not performed [8]. It was not possible to identify another systematic review on this subject among adults. A systematic review and meta-analysis that was published in 2015 [9] performed the search from 2000 to 2013, limiting publications in English, and included studies with different age groups (children, adults, and elderly) and caries assessment in permanent and primary teeth.

In this sense, it is necessary to demonstrate the oral health social determinants [2] by updating the data and analysis by life cycles, as proposed by the World Health Organization—WHO [10]. Another systematic review and meta-analysis that was performed on tooth loss and income with adult individuals showed a connection between tooth loss (evaluated by clinical examination or self-report) and lower income [11]. This report indicated that tooth loss might be associated with other conditions such as periodontal disease and not necessarily to the dental caries process.

Another study updates the later studies on social determinants and caries in adults, with no publication year and language requirements. These studies include new results, comments, and criticisms in an iterative process, enhancing the current scientific knowledge of this subject [12]. New studies can provide important data for a review. This should be valued more than just the change of outcome. Therefore, the update process is not useful when only the primary outcome is changed, however it is when it also maintains the credibility of previous studies [13].

The epidemiological question that was investigated in the present study was whether adults with worse socioeconomic indicators were more affected by dental caries than adults with better socioeconomic indicators?

This study aimed to perform an update of a previous systematic review [8] and to perform a subgroup meta-analysis and meta-regression to evaluate the effect modification of socioeconomic indicators on dental caries in adults. The hypothesis was that adults with worse socioeconomic indicators are more affected by dental caries.

## 2. Materials and Methods

The study protocol was registered in PROSPERO under the reference number: CRD42017074434. This current systematic review and meta-analysis is an update of a review that was previously published by Costa et al. [8]. The Preferred Reporting Items for Systematic review and Meta-Analysis Protocols—PRISMA-P 2015 was used as a reporting guideline [14].

### 2.1. Search Strategy 

The PECO question (P = population; E = exposition or risk factor; C = control; O = outcome) was defined as follows: P—adult individuals, E—worse socioeconomic condition, C—better socioeconomic condition, and O—caries on permanent teeth.

Eight databases were systematically searched: Medline via PubMed (www.pubmed.gov), The Cochrane Library (http://www.cochrane.org/index.htm), including Cochrane database for Systematic Reviews, Database of Abstracts of Reviews of Effectiveness, Cochrane Controlled Trials Register, and Cochrane Review Methodology Database; Web of Science (http://www.isiknowledge.com), Controlled-Trial Database (http://controlled-trial.com), Clinical Trials-US National Institutes of Health (http://www.clinicaltrials.gov), The National Institute for Health and Clinical Excellence (http://www.nice.org.uk) and the Virtual Health Library (Bireme-Latin America; www.bireme.br) as shown in a previous study [8], and Scopus (http://www.scopus.com). The strategies used were identical to the ones that were used in the previous study [8] and in the Scopus which used: ((caries OR Dental Caries [Mesh] OR dental decay OR DMF index [Mesh] OR decayed teeth) AND (socioeconomic factors [Mesh] OR social class [Mesh] OR educational status [Mesh] OR educational level OR socioeconomic condition OR socioeconomic level OR socioeconomic determinants OR social determinants OR income [Mesh] OR poverty [Mesh] OR risk factors [Mesh])). There were no restrictions on language or the year of publication.

The electronic search was performed by three reviewers (CCM, MHNGA, and SMC) and it included articles that were published until March 2017. The studies were entered into the Reference Manager^®^ program. A list was generated for analysis and the selection of titles/abstracts was independently performed by two reviewers (MQCP, SMC) after a calibration exercise with 10% of the studies that were read to determine inter-examiner agreement (Kappa: 0.778). Furthermore, full-texts were retrieved and were independently selected by the same reviewers. Any disagreement was resolved by consensus.

### 2.2. Selection of Studies and Data Extraction

Inclusion criteria were observational studies (cross-sectional, case-control, and cohort) with subjects between 19 and 60 years of age, studies addressing risk factors for dental caries (even if socioeconomic indicators were not the main subject), and studies reporting socioeconomic indicators. The search did not return any trials, although all measures were taken to retrieve them. Reviews were included, and their reference lists were searched in order to find more studies that were not retrieved by the electronic search. However, this process yielded no further studies.

**Figure 1:**
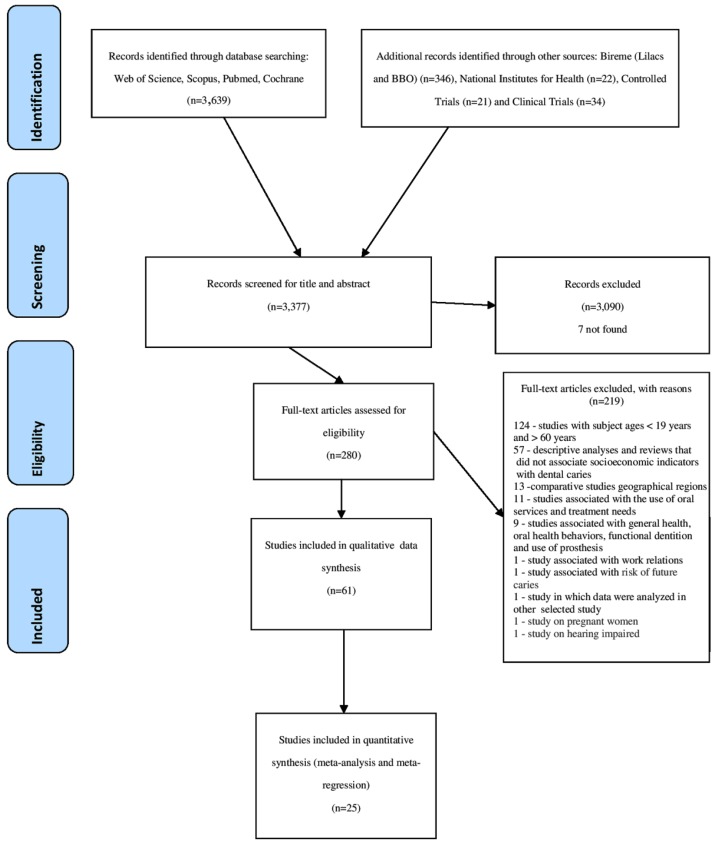
Flowchart of the search process according to PRISMA guidelines.

During this stage, reviews and studies that did not report statistical tests for dental caries and socioeconomic indicators and studies involving individuals that were younger than 19 or older than 60 years old were excluded (Figure 1). Among the studies that were included to update the systematic review, 280 were assessed for eligibility, and 7 could not be found; 6 of them were presented in the previous study [8] in addition to the study of Engstrom and Holmlund, 2011 [15].

The exclusion criteria are shown in Figure 1. We excluded studies without statistical analysis (descriptive studies) and analytical studies that did not present the results of the relationship between dental caries and socioeconomic indicators; comparative studies between geographical regions; studies that were associated with the use of oral services and treatment needs; studies that were associated with general health, oral health behaviors, functional dentition, and the use of prosthesis; studies that were associated with work relationships; studies that were associated with the risk of future caries; studies of pregnant women and of hearing impairment, and studies in which the same data were analyzed in other selected studies. In the latter case, when more than one publication of a single study was verified, those with different cuts of analysis of the socioeconomic indicator were selected for qualitative analysis, however, for quantitative analysis, only the study with the largest population was included.

### 2.3. Data Extraction

Data were extracted by two independent reviewers (Mânia Q. C. Pinto and Simone M. Costa). A data extraction form was developed, and all studies were evaluated with this document. Any disagreement was resolved by consensus. Data were extracted regarding the country, calibration among researchers, place of data collection, language, statistical data treatment, study design, population characteristics, measures that were used for dental caries, and the type of socioeconomic indicators used. The categories and cutoff points of the variables dental caries and socioeconomic indicators were recorded.

### 2.4. Quality Assessment

Two independent reviewers (Mânia Q. C. Pinto and Simone M. Costa) evaluated the quality of the studies using the Newcastle-Ottawa scale (NOS) for cohort studies [16]. Cross-sectional and ecological studies were evaluated using the NOS that had been modified for case-control studies. By using the NOS, the studies were evaluated in items that were organized into three groups: participants selection, groups comparability, and investigation of exposure or outcome. Study quality was rated on a scale from 1 (very poor) to 9 (high). Studies with scores less than 5 were considered as having low methodological quality, scores of 5 to 7 were considered as moderate quality, and those above 7 were considered as high quality [9]. All of the studies that met the selection criteria were included, regardless of the grade that was obtained in the quality assessment. Any disagreement between the reviewers was resolved through discussion and consensus and by consulting a third reviewer (Mânia Q. C. Pinto and Simone M. Costa).

### 2.5. Data Synthesis and Analysis

The authors reported meta-analysis data on DMFT (average of decayed, missing, and filled teeth) as the mean and standard deviation or standard error. The standard error was calculated for those studies that only presented the standard deviation. We extracted the overall DMFT (prevalence) of each study. The overall DMFT of each study was pooled to calculate the overall (ES) DMFT and respective 95% confidence interval (95% CI) for 25 studies that were included in the meta-analysis. If more than one publication that derived from the same sample was retrieved, the study with the largest sample was included in the meta-analysis. STATA software (StataCorp. 2009. Stata Statistical Software: version 11, College Station, TX, USA) was used for the meta-analysis. Heterogeneity was verified by T-squared (Tau^2^ or T^2^) and I-squared (I^2^) tests. The random effect model was used when substantial heterogeneity was found (I^2^ > 50%) [17].

The statistical analysis of the subgroups considered the Human Development Index (HDI), level of schooling, university education, and socioeconomic status (SES). For all of these variables, we considered the country’s overall HDI, overall schooling (in percentage), university education, (overall percentage) and SES (overall percentage) for the whole population of each study and the mean age of the whole population of each study. Each country defined the Human Development Index (HDI) according to the United Nations Development Programme (http://hdr.undp.org/en/composite/HDI). Regarding the subgroup meta-analysis, the countries’ HDI were dichotomized into very high (≥0.800) and high (>0.799), according to the United Nations Development Programme (http://hdr.undp.org/en/composite/HDI).

The subgroups’ level of schooling, university education, SES, and age considered the following cut-off points: Level of schooling: low (≥33.60% of the population with a low level of schooling) and higher (<33.60% of the population with a low level of schooling); Socioeconomic status (SES): low (≥22.4% of the population with a low SES) and higher (<22.4% of the population with a low SES); University education: low (if <33.78% of the population had university level) and higher (≥33.78% of the population had university level); Age: older (if the mean age of the whole population was >33.4 years) and younger (if the mean age of the whole population was ≤33.4 years). Age was reported in 14 studies as the mean estimate of the whole population. The cut-off point of 33.4 years was based on the median for the studies that were included for the variable.

Publication bias was evaluated by the Egger’s test [18,19]. A random-effect meta-regression model was used to explain if the independent variables affected the observed heterogeneity in this meta-analysis. The independent variables that were included in the model were: Continuous variable (HID) and categorical variables (level of schooling, SES, university education, and age). Monte Carlo permutation for adjusted *p*-values was used due to the low number of studies that were included in the meta-regression to decrease the false-positive findings (error type I) [20]. We considered significance as *p* < 0.05. The dependent variable was DMFT (continuous variable).

## 3. Results

### 3.1. Search Outcomes

In total, 4062 potentially relevant records were found. After removing duplicates, 3377 studies were read and 280 were selected for full-text analysis, 61 of which were selected for inclusion in the qualitative synthesis and 25 in the meta-analysis and meta-regression (Figure 1).

### 3.2. Study Characteristics

The systematic review comprised of 45 cross-sectional studies, 14 prospective cohort studies, 1 case-control, and 1 ecological study. No clinical trials were found. Regarding the quality evaluation, study scores are shown in the supplementary Tables S1–S5. Most studies (86.9%) were classified with moderate quality. The studies’ quality ranged from 5 to 9 points, which evidences methodological variability. Lower scores that were obtained in cohort studies that were assessed by the Newcastle-Ottawa quality assessment scale were mainly related to outcome, self-report outcome assessment, and no description of the participants lost for follow up. They were mostly associated with comparability (lack of a multivariate analysis) and exposure (ascertainment of exposure by interview not blinded to the case/control status and non-respondents described) in the cross sectional, case control, and ecological studies.

All of the studies were published between 1975 and 2016 [21,22,23,24,25,26,27,28,29,30,31,32,33,34,35,36,37,38,39,40,41,42,43,44,45,46,47,48,49,50,51,52,53,54,55,56,57,58,59,60,61,62,63,64,65,66,67,68,69,70,71,72,73,74,75,76,77,78,79,80,81]. Twenty new studies were included in this update [26,27,28,29,31,38,39,40,44,45,47,49,50,53,59,61,66,67,79,81]. The language was predominantly English (96.7%). The publications resulted from researches that were conducted in different countries (supplementary Tables S1–S5). The 35–44 years age group was observed in 22 studies (36.0%). Different studies (22.0%) reported the data analysis from a single epidemiological survey, such as: Brennan et al. [21,22,23], Celeste et al. [24,25], Costa et al. [26,27], Zini et al. [28,29], and Holst and Schuller [30,31], however variables differed with regard to categorization.

Only 20 studies (32.8%) reported that the sample group was representative of the population that was studied. All of the prospective studies showed a loss of participants. Among the 61 studies, 33 (54.1%) performed only the bivariate analysis (supplementary Tables S1–S5). Different dental caries measure indexes were identified, with the analysis unit for teeth and surfaces. Several parameters were used, namely, mean, median, quartiles, and others. Criteria that were established by the World Health Organization (WHO) was identified in 34 (55.7%) studies. Only 31 (50.8%) studies described the Kappa index value or the intra-examiner and/or inter-examiner agreement percentage, which varied from 0.61 to 0.98.

Different socioeconomic criteria were considered in the studies, showing considerable diversity among the employed indexes and criteria (supplementary Tables S1–S5): schooling, socioeconomic status (SES), income, government benefits, and community indicators, such as the Gini coefficient.

### 3.3. Eligible Factors for Meta-Analysis and Meta-Regression

The statistical analysis of the subgroups, meta-analysis, and meta-regression involved 25 studies and considered HDI, level of schooling, university education, and SES.

### 3.4. Measured Outcomes Meta-Analysis and Meta-Regression

**Figure 2:**
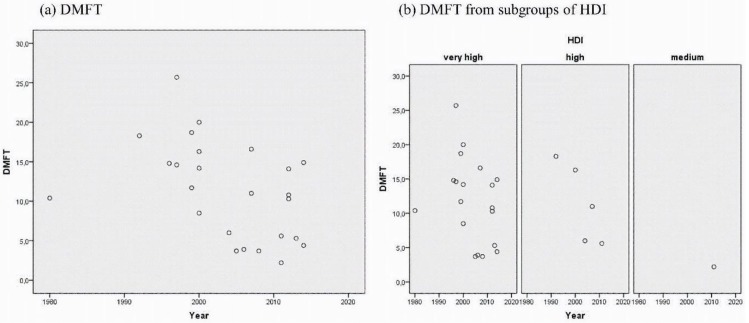
Change of the DMFT (decayed, missing, filled teeth) mean over time. Graphs comparing caries in adults (**a**) total of 25 studies included in the quantitative analysis of caries experience, (**b**) from countries with very high, high, and medium HDI (Human Development Index).

The quantitative analysis evidenced a decreasing trend in the DMFT in the time series, regardless of HDI (Figure 2).

**Figure 3:**
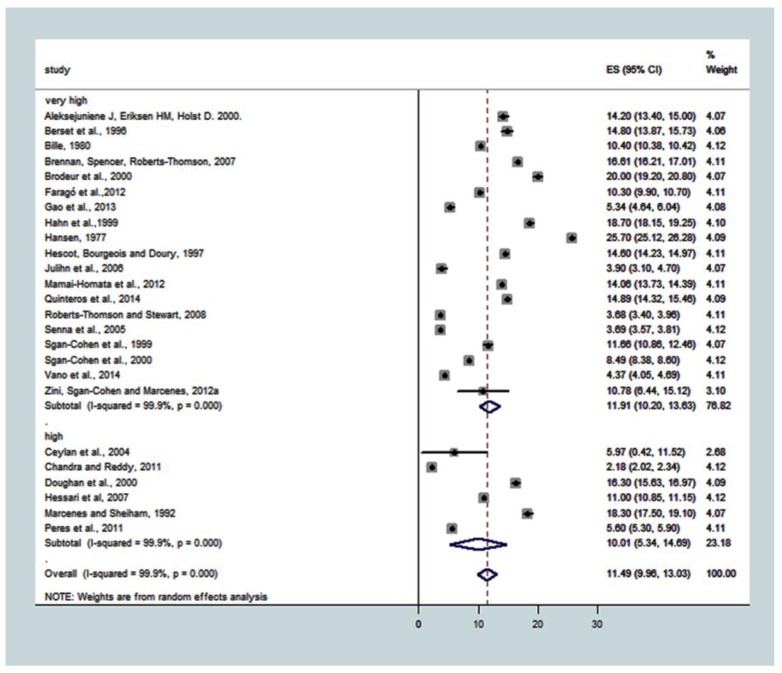
Association between HDI and caries experience (DMFT). Pooled effect estimates from the random-effects meta-analysis are shown. Heterogeneity was assessed using the χ^2^ test and I^2^ statistics. The forest plots are comparing caries in adults from countries with very high compared to high and medium DHI.

HDI categories were very high (*n* = 19) and high (*n* = 6). The overall effect estimate that was common to the groups, which was estimated by the DMFT with a 95% confidence interval (95% CI), was 11.49 (9.96; 13.03). The total random and real heterogeneity for the 25 studies was T^2^ = 14.9162, with real variation I^2^ = 99.9%, *p* < 0.001. For subgroup analysis, the SE (95% CI) of DMFT was 11.91 (10.20; 13.63) for very high HDI and 10.01 (5.34; 14.69) for high HDI. The T^2^ for very high HDI was 14.2633 and 32.9807 for high HDI. DMFT in the very high HDI subgroup was similar to the high HDI (Figure 3).

**Figure 4:**
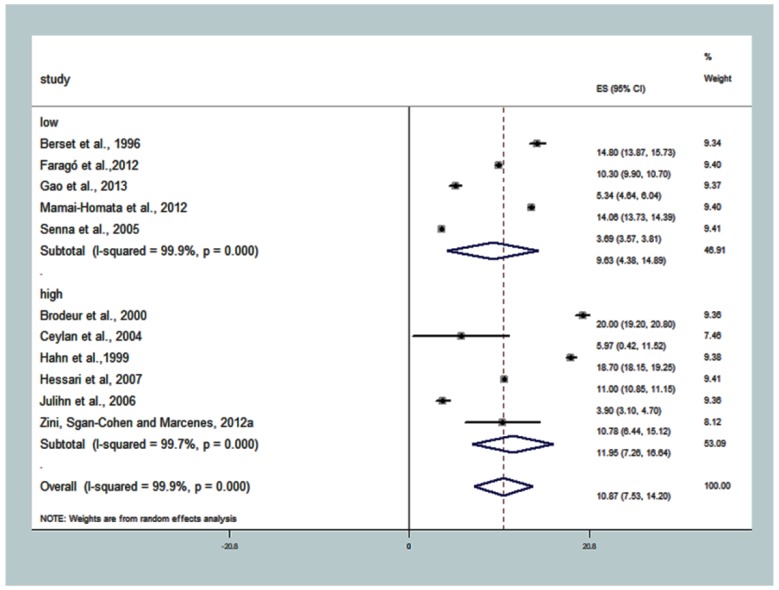
Association between subgroup ‘low level of schooling’ and caries experience (DMFT). The pooled effect estimates from the random-effects meta-analysis are shown. Heterogeneity was assessed using the χ^2^ test and I^2^ statistics. The forest plots are comparing caries in adults with ≥33.60% of the population with a low level of schooling-low compared with <33.60% of the population with a low education-higher.

The overall summary DMFT for ‘low level of schooling’ was 10.87 (7.53; 14.20), T^2^ = 30.7610, I^2^ = 99.9%, *p* < 0.001. The effect estimate for ‘high’ level of schooling (where ≤33.6% of people had low schooling) had DMFT = 11.95 (7.26; 16.64), T^2^ = 32.3987, I^2^ = 99.7%, *p* < 0.001. In the subgroup with studies with the highest percentage of the population with low schooling (‘low’ subgroup), T^2^ was 35.8766, that is, there was greater total heterogeneity among the studies (supplementary Table S1; Figure 4), and DMFT = 9.63 (4.38; 14.89).

**Figure 5:**
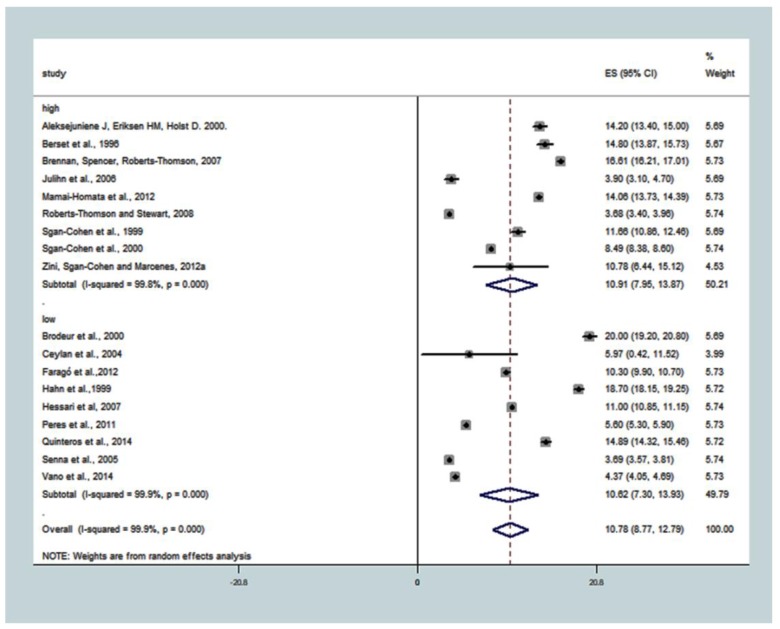
Association between the subgroup ‘university’ and caries experience (DMFT). The pooled effect estimates from the random-effects meta-analysis are shown. Heterogeneity was assessed using the χ^2^ test and I^2^ statistics. The forest plots are comparing caries in adults with <33.78% of the population with university-low compared with ≥33.78% of the population with a university-higher.

The DMFT summary measure for the subgroup ‘university’ was 10.78 (8.77; 12.79), T^2^ = 18.2688, I^2^ = 99.9%, *p* < 0.001. The DMFT common effect size in a subgroup with a high proportion of people with university level was 10.91 (7.95; 13.87), T^2^ = 19.9362, I^2^ = 99.8%, *p* < 0.001, while in the subgroup with a lower proportion of people with university education (low), it was 10.62 (7.30; 13.93), T^2^ = 24.9764, I^2^ = 99.9%, *p* < 0.001 (Figure 5).

**Figure 6:**
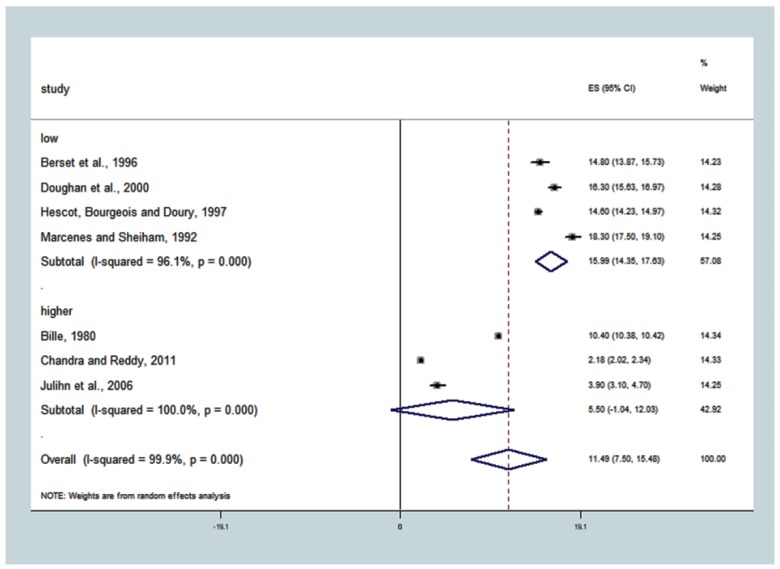
Association between ‘low socioeconomic status-SES’ and caries experience (DMFT). The pooled effect estimates from the random-effects meta-analysis are shown. Heterogeneity was assessed using the χ^2^ test and I^2^ statistics. The forest plots comparing caries in adults with ≥22.4% of the population had low socioeconomic status SES-low compared with <22.4% of the population with low SES-higher.

For the subgroup of studies with populations with more people with ‘low’ SES (≥22.4%), the DMFT was higher 15.99 (14.36; 17.63), T^2^ = 2.6810, I^2^ = 96.1%, *p* < 0.001. For the subgroup where studies had populations with ‘higher’ SES, the DMFT was lower 5.50 (−1.04; 23.03). The overall estimate for SES, in general, showed DMFT to be equal to 11.49 (7.50; 15.48), T^2^ = 28.8521, with significant heterogeneity between the studies, I^2^ = 99.9%, *p* < 0.0001 (Figure 6). All of the covariates evidenced a variation in DMFT that was attributed to the real studies heterogeneity, with a small percentage of random variable explanation, from 0.1% to 3.9%.

**Figure 7:**
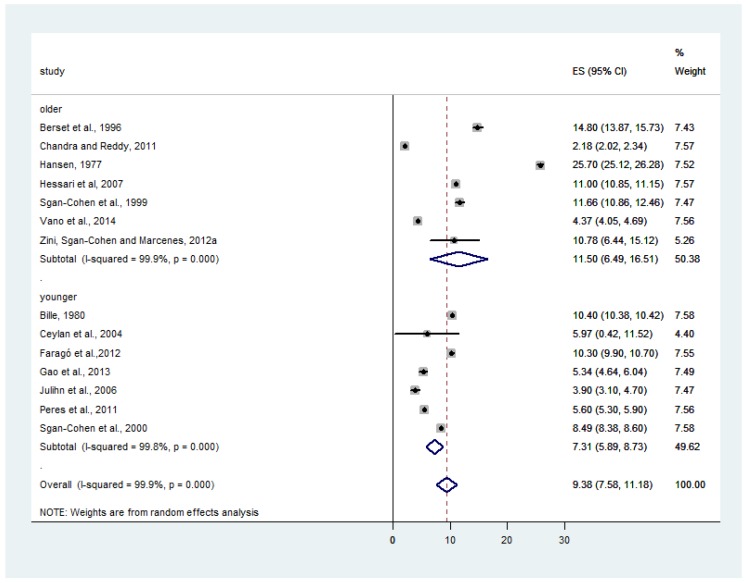
Association between ‘age’ and caries experience (DMFT). The pooled effect estimates from the random-effects meta-analysis are shown. Heterogeneity was assessed using the χ^2^ test and I^2^ statistics. The forest plots are comparing caries in studies with older population (>33.4 years) compared with studies with younger population (≤33.4 years)**.**

The subgroup of studies with an older population (mean age >33.4 years) had higher DMFT (11.50; 95%CI: 6.49; 16.51), T^2^ = 45.0099, I^2^ = 99.9%, *p* < 0.001. For the subgroup where studies had a younger population (≤33.4 years), the DMFT was lower (7.31; 95%CI: 5.89; 8.73), T^2^ = 3.2631, I^2^ = 99.8%, *p* < 0.001. The overall effect estimate for age in the 14 studies that were included showed a DMFT equal to 9.38 (7.58; 11.18), T^2^ = 11.1319, I^2^ = 99.9%, *p* < 0.0001 (Figure 7).

In the non-adjusted meta-regression model, the multiple factors effect analysis on the results of heterogeneity showed no DMFT association with HDI, higher education, low level of schooling, and age. The studies with a higher proportion of populations with a low SES showed higher DMFT (*p* = 0.050). The variation between the studies of the SES covariate against the DMFT can be explained at a level of 76.25%, and the variation within the studies is explained at a level of 23.75% (Table 1).

The adjusted model 2 better explained the variation in DMFT between the studies as can be observed by the lower Tau value (10.56) between the two adjusted models. The increase of one unit of SES level was associated with an increase in 10.35 units in DMFT, *p* = 0.050 (Table 1). Therefore, it is implied that there is an increase of 10 more teeth in the DMFT index in studies with >22% of people with low SES.

## 4. Discussion

This is an update of a previous systematic review, reporting the caries experience (DMFT mean) in adults from socioeconomic indicators. In our previous review which only considered qualitative data analysis, 41 studies were included [8]. In the current review, another 20 studies were added which met the inclusion criteria, totalizing 61 in the qualitative analysis. Of these 61 articles, 25 met the requirements for the quantitative data analysis, meaning they were submitted to the meta-regression. The paper from Schwendicke et al. [9] included the evaluation of decayed teeth in children, adults and, the elderly, and there was no age limit, which explains why it had the greatest number of articles, since the evaluation included DMFT and dmft (permanent teeth and deciduous teeth) [9]. Our study involved only adults, that is, a specific life cycle.

This study’s inclusion criterion was the 19–60 years age group, a strategy that was used to expand the search and selection of dental caries studies in adults. Another systematic review and meta-analysis of dental loss and income used the age group from 18 to 60 years old, which is similar to the one that was used in the current study [11]. The 35–44 years age group that was recommended by the WHO [10] was only used by 22 references.

The quality of the studies ranged from moderate to high quality (five to nine points), which shows methodological variability. The use of scales for quality assessment has limitations [82], and the use of the scale and weight criteria can be very subjective among reviewers [8].

The period of 1975–1999 returned 14 publications, an average of 1.56 references per year. Most of the publications (*n* = 47) were concentrated in the period from 2000 to 2016, with an average of 2.93 references per year. Cross-sectional studies were the most common types of studies. Only one case-control study was found, which shows the need for future studies with this design since it provides greater scientific evidence than cross-sectional studies. The lack of participants in the oral exam was another negative aspect. Losses due to follow up were significant in the cohort studies.

The evaluation of the studies that were published between 1975 and 2016 suggests a reduction in the DMFT value over time. However, it is unlikely that this reduction has affected all of the geographical regions and their populations since reports disagree with the literature on the lack of improved oral health conditions in the last few decades [3].

The DMFT metassumarized measurement was 11.49 (9.96–13.03), which reflects a moderate dental caries level [83]. The heterogeneity was high, which requires careful presentation and interpretation of the DMFT overall estimate. The presence of high heterogeneity means that there is a strong effect modification by the third variable or, in other words, that there is an effect of the confounders. This is particularly common in the meta-analysis of prevalence data of observational studies. For this reason, we performed a meta-regression to evaluate the effect of other variables in the heterogeneity of the meta-analysis [84].

The higher caries experience was associated with low SES which, in other words, means people who are deprived of economic and educational resources. This shows that low SES can be considered as a marker for increased risk of dental caries [85]. The high prevalence of oral diseases requires global public health policy decisions from measurable goals. The 2015 Global Burden of Disease recognized oral diseases as a global public health challenge, with a 64% increase in untreated people [3], which requires monitoring and a reduction in health inequalities [7], because it is a fundamental right regardless of people’s SES.

The meta-regression showed that studies that were conducted with older adults (>33.4 years) had higher values of DMFT (effect estimate: 11.50) compared with studies that were conducted with younger adults (aged from 19 to 33.4 years; DMFT = 7.31). A recent longitudinal study showed that socioeconomic disparities in oral health vary by age. The outcome ‘teeth not in good condition’ was self-reported and was analyzed according to age and socioeconomic group (‘never poor’ versus ‘poor at least once in life’). The prevalence differences (95%CI) of the oral health inequalities among individuals aged 15–24 years was 5.7% (1.3 to 10.1), while among adults aged from 45 to 54 years, the prevalence of oral health inequalities was also higher (12.6%; 95% CI: 8.7–16.5) [86].

In the meta-regression quantitative analysis, populations with higher percentages of people with a low SES had a higher DMFT, both in the unadjusted model (*p* = 0.031) and in the HDI adjusted model, although the level of statistical significance was in the borderline range (*p* = 0.050). The variables that evaluated SES were presented in different formats, such as social class, socioeconomic status, parent’s SES, cumulative effects of the income and educational level, SES by ABA-ABIPEME (resources and educational level), and SES trajectory (early childhood SES to age-26-years SES). The categories of analysis were presented between two to five comparison groups.

Social disadvantage can be measured in several ways [5,87] and is associated with negative impacts on the oral health of a population. The SES supports three major determinants: health behaviors, environmental exposures, and health care. A study defined the importance of social determinants in oral health inequality, and different pathways have been presented for intervention on the determinants during the life cycle. Among the interventions that have been proposed are actions to: eliminate the sources of inequalities in oral health; to protect oral health as a human right through international and national policies; to strengthen intersectoral strategies for poverty reduction; supporting scientific research on the health social determinants; interact communities with public health managers and researchers; support community actions to promote oral health; eliminate barriers of access to oral health care; and to promote literacy in oral health, with the dissemination disease prevention value [87].

For all of the evaluated covariates, the DMFT variation was attributed to the studies’ real heterogeneity, with I^2^ between 96.1% and 99.9%. However, it is normal to find statistical heterogeneity in non-randomized and prevalence studies. Although bias observational studies lack randomization, it is justified to include them more and more in the systematic analyzes when tests can be considered unviable or unethical [88]. Therefore, methods of causal inference from observational data have increased relevance in clinical medicine and public health research [89].

The meta-regression found no association between DMFT and HDI, a lower proportion of people with higher education, a higher proportion of people with a low schooling level, and age. Despite the importance of education as a measure of socioeconomic position, measuring years of education or levels of schooling may not contain data on the education experience quality. Therefore, if the education variable is used only as a socioeconomic position indicator, it will become less important [5].

The higher proportion of people with low socioeconomic status was associated with higher DMFT, although the level of statistical significance was in the borderline range (*p* = 0.050). The magnitude of this association reinforces the need for reducing and tackling oral health disparities [5,86].

This study has limitations, such as not providing a meta-analysis for all of the qualitative synthesis social determinants of the Supplementary Table S1–S5 due to the lack of data homogeneity and DMFT unavailability for the entire population. Most studies are cross-sectional and are not risk-predictive. The search was conducted in March 2017 and new studies may have been published. There was a lack of consensus on the social determinants’ categorization, education level, occupation, income, and SES were defined and classified differently in the studies that were analyzed. The results refer to studies with adults aged 19–60 years and, therefore, should not be generalized to elderly populations. High heterogeneity was detected between the studies (I^2^ > 90.0%).

We recommend the following actions:

- Performing investigations with the age bracket suggested by WHO for adults;
- Using standardized measures to evaluate the effect of socioeconomic indicators on caries;
- Conducting cohort and case-control studies;
- Building an SES evaluation standard that fits different country situations, since it can be evaluated by income and education, however on the other hand, it can also be evaluated by other physical, financial, and organizational productive resources [5];
- Incorporating multivariate analyzes to verify confounding elements; and
- Carrying out studies with population representativeness and a description of the disease for the entire study population.

## 5. Conclusions

In conclusion, the present study findings provide evidence that populations with the highest proportions of people with a low SES are associated with a greater severity of dental caries in adults. Public health managers and dentistry professionals should propose actions to reduce oral health inequalities. It is necessary to step up efforts in order to give people with lower SES more benefit from caries prevention products and to increase their access to dental services. In addition, health literacy should be working on empowering the subjects and making the right decisions to promote oral health.

## Figures and Tables

**Table 1:** Meta-regression for decayed, missing, and filled teeth and covariates.

Models	R^2^	Tau^2^	Coefficient	Standard Error	*p*-Value ^†^
Unadjusted models					
Human Development Index—HDI (quantitative)	2.85%	36.39	20.17878	15.26451	0.180
University (<33.78% of the population with a university)	−6.22%	30.11	−0.3066788	2.615528	0.919
Education (>33.60% of the population with low education)	−5.55%	34.34	−2.301079	3.596737	0.546
Socioeconomic Status—SES (>22.4% of the population with low SES)	76.25%	9.302	10.49365	2.340803	0.031
Age (older population—mean age of the population >33.4 years)	6.41%	33.25	4.324884	3.121433	0.197
Adjusted models					
Model 1 *					
HDI (quantitative)	100.0%	0.0	−42.94101	142.3087	0.956
University degree (<33.78%)			−4.132903	17.93963	0.974
Education (>33.60% with low education)			7.205453	7.012035	0.687
Age (older population—mean age of the population >33.4 years)			5.248158	1.843047	0.282
Model 2 **					
HDI (quantitative)	73.04%	10.56	6.901906	11.08584	0.832
SES (>22.4% of the population had low SES)			10.35391	2.503207	0.050

R^2^ = proportion of the between-study variance explained, Tau^2^ = the estimative of between-study variance, coefficient, standard error of DMFT, and *p* value. ^†^ adjusted *p*-value by Monte Carlo permutation. * 6 observations were included in the model. ** 7 observations were included in the model.

## Supplementary Materials

The following are available online at http://www.mdpi.com/1660-4601/15/8/1775/s1, Table S1: Study characteristics and results reported of the indicator income, Table S2: Study characteristics and results reported of the indicator education, Table S3: Study characteristics and results reported of the occupational status, Table S4: Study characteristics and results reported of the socioeconomic status, Table S5: Study characteristics and results reported of the collective indicators and other.
