# Supplementary material for: Socioeconomic Factors and Caries in People between 19 and 60 Years of Age: An Update of a Systematic Review and Meta-Analysis of Observational Studies
Source: Int J Environ Res Public Health. 2018 Aug 18;15(8):1775. doi: 10.3390/ijerph15081775 (PMC6121598; doi:10.3390/ijerph15081775)
Supplement: Supplementary file 1 [file ijerph-15-01775-s001.pdf]

International Journal of
Environmental Research and Public Health

MDPI

Supplementary Table S1. Study characteristics and results reported of the indicator income.

**Authors, year, country**	**Study type**	**Sample in the analysis**	**Caries index**	**Effect measure**	**Income: categories of analysis Socioeconomic parameter (reference*)**	**Group with higher caries index**	**Bivariate analysis p value**	**Multivariate analysis *p* value**	**NOS**
Berset et al. (1996), Norway	Cross sectional	119	Decayed surfaces -DS	Mean (SD)	economy: satisfied, minimun problems, major problems	major problems	4.45 (4.5) p < 0.01	does not describe values multiple regression	7
Bjertness et al. (1992), Norway	Cohort	81	Decayed teeth-DT	Mean (SD)	satisfaction with own finances: unsatisfied, satisfied	satisfied	1.24 (0.43) p > 0.05	not available	8
Brenan and Spencer (2014) Australia	Life course Caries - 30 years old	435	DMFT	Mean (SD) RR (95%CI)	health card status, age 13 years: card holder, no card* (family was covered by a government health card at age 13 years for disadvantaged group, example unemployed)	card holder	4.88 (0.38) p > 0.05	RR= 0.9 (0.7, 1.1) p > 0.05	6
			Decayed teeth-DT	Mean (SD), RR (95%CI)	health card status, age 13 years: card holder, no card* (family was covered by a government health card at age 13 years for disadvantaged group, example unemployed)	card holder	1.11 (0.20) p < 0.01	RR= 2.2 (1.4, 3.3) p < 0.01	
Brenan and Spencer (2015) Australia	Life course Caries - 30 years old	411	DMFT	Mean (SD), RR (95%CI)	critical period - family income at age 13: low income, higher income*	low income	4.82 (0.44) p > 0.05	RR= 1.0 (0.8, 1.2) p > 0.05	6
			DMFT	Mean (SD), RR (95%CI)	cumulative risk - family income at age 13, own income at age 30 years and education and occupation: lower risk*, moderate risk, higher risk	higher risk	5.33 (0.42) p > 0.05	RR= 1.2 (0.9, 1.4) p > 0.05	
			DMFT	Mean (SD), RR (95%CI)	social mobility - income at age 13 and 30 years: advantaged, upwardly mobile*, middle, downwardly mobile, disadvantaged	disadvantaaged	6.53 (0.85) p < 0.01	RR= 1.5 (1.1, 2.1) p < 0.01	
			Decayed teeth-DT	Mean (SD), RR (95%CI)	critical period - family income at age 13: low income, higher income*	low income	0.82 (0.19) p > 0.05	RR= 1.01 (0.7, 1.5) p > 0.05	
			Decayed teeth-DT	Mean (SD), RR (95%CI)	cumulative risk - family income at age 13, own income at age 30 years and education and occupation: lower risk*, moderate risk, higher risk	higher risk	1.05 (0.20) p < 0.01	RR= 1.6 (1.1, 2.4) p < 0.05	
			Decayed teeth-DT	Mean (SD), RR (95%CI)	social mobility - income at age 13 and 30 years: advantaged, upwardly mobile*, middle, downwardly mobile, disadvantaged	disadvantaged	1.53 (0.43) p < 0.01	RR= 3.1 (1.7, 5.6) p < 0.01	

(continued next page)

Supplementary Table S1. (continued)

Authors, year, country	Study type	Sample in the analysis	Caries index	Effect measure	Income: categories of analysis Socioeconomic parameter (reference*)	Group with higher caries index	Bivariate analysis p value	Multivariate analysis p value	NOS
Brennan, Spencer and Roberts-Thomson (2007), Australia	Cross sectional	709	Decayed teeth - DT	Mean (SD), Beta	family income: ≥ $80 000; <$80 000*	< $80 000	0.48 (0.05) p < 0.01	≥ $80 000 Beta= -0.15 p > 0.05	7
			DMFT	Mean (SD)	family income: ≥ $80 000; < $80 000*	< $80 000	17.01 (0.24) p < 0.01	≥ $80 000 Beta= -1.35 p < 0.01	
Brennan, Spencer and Roberts-Thomson (2010), Australia	Cross sectional	709	Decayed teeth - DT	Mean (SD), Beta (SE)	family income: ≥ $80 000, < $80 000*	< $80 000	0.5 (0.05) p < 0.01	≥ $80 000 Beta= -0.27 (0.09) p < 0.01	7
			DMFT	Mean (SD), Beta (SE)	family income: ≥ $80 000, < $80 000*	< $80 000	17.0 (0.2) p < 0.01	≥ $80 000 Beta= -1.21 (0.49) p < 0.01	
Brennan, Spencer and Roberts-Thomson (2011), Australia	Cross sectional	709	Decayed teeth - DT	Mean (SD)	family income: < AU$30,000, AU$30,000–$60,000, > AU$60,000	< AU$30,000	0.8 (0.13) p < 0.01	the multivariate model of DT showed significant effects p < 0.0001	7
			DMFT	Mean (SD)	family income: < AU$30,000, AU$30,000–$60,000, > AU$60,000	< AU$30,000	17.1 (0.45) p < 0.01	not available	
Brodeur et al. (2000), Canada	Cross sectional	2,110	Decayed surfaces - DS	Mean	family income: < $30,000, $30,000 to $59,999, ≥ $60,000*	< $30,000	2.6 p < 0.05	not available	7
			DS (≤3, ≥4)	Odds ratio-OR (95%CI)	family income: < $30,000, $30,000 to $59,999, ≥ $60,000*	< $30,000	not available	3.8 (2.19,6.48) p < 0.05	

(continued next page)

Supplementary Table S1. (continued)

**Authors, year, country**	**Study type**	**Sample in the analysis**	**Caries index**	**Effect measure**	**Income: categories of analysis Socioeconomic parameter (reference*)**	**Group with higher caries index**	**Bivariate analysis p value**	**Multivariate analysis p value**	**NOS**
Celeste et al. (2011), Brazil	Cross sectional	12,154	Decayed teeth - DT	Mean (SD)	family income (based on minimum wage): up to 1/2; 1/2 to 1; 1 to 2; 2 to 3; +3	up to 1/2	4.26 (4.53) $p < 0.001$	municipal income effect	7
Ceylan et al. (2004), Turkey	Cross sectional	2766	DMFT	Mean	income level: 0-49 million TL, 50-99, 100-199, ≥200 million TL	≥ 200 million TL	7.35 $p < 0.001$	not available	
			DMFT	Correlation coefficient R	monthly income per capita in Turkish liras (TL)	positive correlation	0.080 $p < 0.01$	not available	
			Decayed teeth - DT	Correlation coefficient R	monthly income per capita in Turkish liras (TL)	negative correlation	-0.082 $p < 0.01$	not available	
Costa et al. (2012), Brazil	Cross sectional	1138	DMFT (<14, ≥14)	Prevalence ratio -PR	montly family income: > 600 dólares*; ≤ 600 dolares	≤ 600 dolares	1.09 $p < 0.05$	1.11 $p < 0.05$	7
Costa et al. (2013), Brazil	Case-control	360 180 case 180 control	DMFT (<14, ≥14)	Odds ratio OR (95%CI)	montly family income: > US$600*, ≤ US$600	≤ US$600	1.7 (1.1,2.6) $p < 0.05$	2.2 (1.3, 3.9) $p < 0.05$	8
Divaris et al. (2012), North Carollina	Cohort	215	Caries increment third molar	Rate ratio (95%CI), incidence rate ratio	income: <$20,000*, $20,000 - $39,999, $40,000 - $60,000, >$60,000	< $20,000	> $60,000 0.54 (0.29, 0.99) $p < 0.05$	incidence rate ratio= 1.04 for 1 unit covariate increase	8
Do L. (2012), Australia	Life course	1,221	DMFS	Mean (95%CI)	family income: < 36400 A$, 36400 - < 2000 A$, 52200 – < 78000 A$, ≥78000 A$	< 36400 A$	6.62 (4.99, 8.26) $p < 0.05$	not available	7
Geyer et al. (2010), Germany	Cross sectional	925	DMFT (≤21, >21)	OR (95%CI)	income: highest*, second highest, intermediate, second lowest and lowest	lowest	3.74 (1.66, 8.46) $p < 0.05$	2.34 (1.00, 5.55) $p > 0.05$	7

(continued next page)

Supplementary Table S1. (continued)

Authors, year, country	Study type	Sample in the analysis	Caries index	Effect measure	Income: categories of analysis Socioeconomic parameter (reference*)	Group with higher caries index	Bivariate analysis p value	Multivariate analysis p value	NOS
Gilbert et al. (2001), USA	Cohort	696	Decayed or filled root surface	Percentage	annual family income: < $20,000; ≥ $20,000	< $20,000	p<0.05	not available	8
Guiotoku et al. (2012), Brazil	Cross sectional	6,918	DMFT	Correlation coefficient	family income	positive correlation	0.1 p > 0.05	not available	5
Lee et al. (2012), Korean	Cross sectional	4,053	DMFT ≥ 1 19-34 years	Odds ratio OR (95%CI)	monthly family income (thousand Korean won): ≥ 1500*, 1000-1490, 500-990, < 500	< 500	1.03 (0.59, 1.79) p > 0.05	not available	5
			DMFT ≥ 1 35-44 years	Odds ratio OR (95%CI)	monthly family income (thousand Korean won): ≥ 1500*, 1000-1490, 500-990, < 500	1,000 - 1,490	1.23 (0.80, 1.90) p > 0.05	not available	
			DMFT ≥ 1 45-54 years	Odds ratio OR (95%CI)	monthly family income (thousand Korean won): ≥ 1500*, 1000-1490, 500-990, < 500	1,000 - 1,490	1.04 (0.56, 1.94) p > 0.05	not available	
Mamai-Homata et al. (2012), Grécia	Cross sectional	1,184	Decayed teeth - DT	Mean,	monthly income: ≤ 590E*, 591-1760E, ≥ 1761E	≤ 590E	2.24 p > 0.05	not available	7
			DMFT	Mean (SD)	monthly income: ≤ 590E*, 591-1760E, ≥ 1761E	≤ 590E	14.65 (5.89) p > 0.05	not available	
			DMFS	Mean (SD), OR (95%CI)	monthly income: ≤ 590E*, 591-1760E, ≥ 1761E	≤ 590E	49.29 (26.83) p < 0.05	≥ 1761E OR= 0.835 (0.357, 1.957) p > 0.05	
			Decayed surfaces – DS	Mean	monthly income: ≤ 590E*, 591-1760E, ≥1761E	≤ 590E	5.26 p > 0.05	not available	
			RDFS	Mean (SD), OR (95%CI)	monthly income: ≤ 590E*, 591-1760E, ≥1761E	≤ 590E	0.36 (1.36) p > 0.05	≥ 1761E OR= 0.375 (0.080, 1.750) p > 0.05	

(continued next page)

Supplementary Table S1. (continued)

**Authors, year, country**	**Study type**	**Sample in the analysis**	**Caries index**	**Effect measure**	**Income: categories of analysis Socioeconomic parameter (reference*)**	**Group with higher caries index**	**Bivariate analysis p value**	**Multivariate analysis p value**	**NOS**
Nikias et al. (1975), USA	Cross sectional	991	Decayed teeth - DT	Mean	status: poverty and non-poverty	poverty	1.6<br>not given p value	not available	5
			DT: 0, 1-2, ≥3	Percentage	status: poverty and non-poverty	poverty	$p < 0.05$	not available	
Peres et al. (2011), Brazil	Cohort	720	DT	Mean	family income (minimum-wages): ≤ 1, 1.1-3.0, 3.1-6.0, 6.1-10.0, > 10.0	less family income	does not describe values - $p < 0.001$	not available	7
			DMFT	Mean	family income (minimum-wages): ≤ 1, 1.1-3.0, 3.1-6.0, 6.1-10.0, > 10.0	higher family income	does not describe values - $p > 0.05$	not available	
Roberts-Thomson and Stewart (2008), Australia	Cross sectional	644	DMFS	Mean (SD)	income: < $20 000, ≥ $20 000	< $20 000	6.26 (9.26)<br>$p > 0.05$	not available	7
			Decayed surfaces - DS	Mean (SD)	income: < $20 000, ≥ $20 000	< $20 000	0.94 (3.18)<br>$p > 0.05$	Linear regression no including variable income	
Skudutyte-Rysstad et al (2009), Norway	Cross sectional	149	DS: ≥ 2, < 2	Percentage, OR (95%CI)	familly income (NOK/year): ≤ 299,000 (low),300,000–599,000 (medium) and ≥ 600,000 (high)	low	$p < 0.05$	$p > 0.05$<br>2.1 (0.8, 5.8)	6
Zini et al. (2012)a, Israel	Cross sectional	254	DMFT	Mean (95%CI)	income: low; salary	salary	11.05 (10.29, 11.80)<br>$p > 0.05$	Logistic regression no including variable income	7
			DT	Mean (95%CI)	income: low; salary	salary	9.96 (0.78, 1.14)<br>$p > 0.05$	Logistic regression no including variable income	

Risk of bias was assessed using the Newcastle-Ottawa (NOS) for observacional studies (Wells et al. 2009) with scores for summarizing the multitude data. DMFT decayed missing filled teeth; DT decayed teeth; DS/DFS decayed (filled) surfaces; DMFS decayed missing filled surfaces; RDFS, RDS decayed (filled) surfaces root; DF decayed root; Mean (SD) standard deviation; (SE) standard error; RR risk ratio; 95%CI confidence interval; OR odds ratio; PR prevalence rate; R correlation coefficient.

Supplementary Table S2. Study characteristics and results reported of the indicator education.

**Authors, year, country**	**Study type**	**Sample in the analysis**	**Caries index**	**Effect measure**	**Education: categories of analysis Socioeconomic parameter (reference*)**	**Group with higher caries index**	**Bivariate analysis p value**	**Multivariate analysis p value**	**NOS**
Aleksejuniene, Eriksen and Holst (2000), Lithuania	Cross sectional	382	DMFT	Mean (SD)	years of education: < 11, 11-13, > 13	> 13 years	14.8 (5.9) p > 0.05	not available	6
			Decayed surfaces-DS	Mean (SD)	years of education: < 11, 11-13, > 13	< 11 years	10.4 (11.5) p < 0.01	not available	
Badel et al. (2006), Croatia	Cross sectional	248	DMFT	Median	education: primary, secondary, university	primary	7.0 p > 0.05	not available	6
			Decayed teeth-DT quartile cutoffs: 25, 50, 75%	Median	education: primary, secondary, university	Primary = Secondary	2 p < 0.05	not available	
Berset et al. (1996), Norway	Cross sectional	120	DS	Mean (SD)	years of education: < 12, > 12	< 12 years	3.1 (3.8) p < 0.05	does not describe values multiple regression	7
Bjertness et al. (1992), Norway	Cohort	81	DT	Mean (SD)	years of education: ≤ 10 , > 10	≤ 10 years	1.27 (0.452) p > 0.05	Multivariate model no including education	8
Brennan, Spencer and Roberts-Thomson (2007), Australia	Cross sectional	709	DMFT	Mean (SE), Beta	education: diploma or degree, primary/secondary/certificate*	primary/secondary/certificate	17.36 (0.27) p < 0.01	Diploma Beta= -1.27 p < 0.01	7
			DT	Mean (SE), Beta	education: diploma or degree, primary/secondary/certificate*	primary/secondary/certificate	0.53 (0.06) p < 0.01	Diploma Beta= -0.12 p > 0.05	

(continued next page)

Supplementary Table S2. (continued).

Authors, year, country	Study type	Sample in the analysis	Caries index	Effect measure	Education: categories of analysis Socioeconomic parameter (reference*)	Group with higher caries index	Bivariate analysis p value	Multivariate analysis p value	NOS
Brennan, Spencer and Roberts-Thomson (2010), Australia	Cross sectional	709	DMFT	Mean (SE), Beta (SE)	education: tertiary, secondary*	secondary	17.4 (0.3) $p > 0.05$	tertiary Beta= -1.35 (0.43) $p < 0.01$	7
			Decayed teeth-DT	Mean (SE), Beta (SE)	education: tertiary, secondary*	secondary	0.5 (0.06) $p < 0.05$	tertiary Beta= -0.25 (0.08) $p < 0.01$	
Brodeur et al. (2000), Canada	Cross sectional	2,110	Decayed surfaces-DS (≤3, ≥4)	Odds ratio OR (95%CI)	education: primaryl/high school, vocational training/college, university*	primary/high school	no test	1.2 (0.75, 1.81) $p > 0.05$	7
Ceylan et al. (2004), Turkey	Cross sectional	2,766	DMFT	Mean, Correlation coefficient R	years of schooling: illiterate, 1-8, 9-11, ≥ 12	illiterate, negative correlation	7.71 $p < 0.001$ R= -0.031 $p > 0.05$	not available	8
			DMFT	Mean, Correlation coefficient R	mother's education: illiterate, literate	illiterate, negative correlation	6.20 $p < 0.001$ R= -0.036 $p > 0.05$	not available	
			DMFT	Mean, Correlation coefficient R	father's education: illiterate, 1-8 years, ≥ 9 years	illiterate, positive correlation	6.16 $p > 0.05$ R= 0.004 $p > 0.05$	not available	
			DT	Correlation coefficient R	years of schooling: illiterate, 1-8, 9-11, ≥ 12	negative correlation	R= -0.181 $p < 0.01$	not available	
			DT	Correlation coefficient R	mother's education: illiterate, literate	negative correlation	R= -0.074 $p < 0.01$	not available	
			DT	Correlation coefficient R	father's education: illiterate, 1-8 years, ≥ 9 years	negative correlation	R= -0.029 $p > 0.05$	not available	

(continued next page)

Supplementary Table S2. (continued).

Authors, year, country	Study type	Sample in the analysis	Caries index	Effect measure	Education: categories of analysis Socioeconomic parameter (reference*)	Group with higher caries index	Bivariate analysis p value	Multivariate analysis p value	NOS
Costa et al. (2012), Brazil	Cross sectional	1133	DMFT (< 14, ≥ 14)	Prevalence ratio (95%CI)	education: university*, non-university	non-university	1.17 (0.95, 1.46) $p > 0.05$	multivariate model no including variable education	7
Costa et al. (2013), Brazil	Case-control	360 180 case and 180 control	DMFT (< 14, ≥ 14)	Odds ratio OR (95%CI)	education literate*, illiterate	literate	Illiterate 0.2 (0.1, 1.2) $p > 0.05$	multivariate model no including variable education	8
Divaris et al. (2012), North Carolina	Cohort	215	Caries increment third molar	Rate ratio (95%CI), incidence rate ratio	education: some college or less, college*, post college	some college or less	2.17 (1.32, 3.58) $p < 0.05$	incidence rate ratio=0.76 - for 1 unit covariate increase	8
Do L. (2012), Australia	Life course	1,221	DMFS	Mean (95%CI)	education: school only, vocational training, tertiary or higher	vocational training	5.77 (5.01, 6.53) $p < 0.05$	multivariate model no including variable education	7
Edman et al. (2016), Sweden	Cross sectional	Analysis per year/age old 2003/35 n=284	Decayed surfaces – DS (=0, ≥1)	Percentage	education: high, low	low	$p = 0.052$	multivariate with inclusion > 60 years, so it was not considered.	6
		2003/50 n=347	DS (=0, ≥1)	Percentage	education: high, low	low	$p < 0.05$	multivariate with inclusion > 60 years	
		2008/35 n=207	DS (=0, ≥1)	Percentage	education: high, low	low	$p < 0.05$	multivariate with inclusion > 60 years	
		2008/50 n=246	DS (=0, ≥1)	Percentage	education: high, low	low	$p > 0.05$	multivariate with inclusion > 60 years	
		2013/35 n=198	DS (=0, ≥1)	Percentage	education: high, low	low	$p < 0.05$	multivariate with inclusion > 60 years	
		2013/50 n=335	DS (=0, ≥1)	Percentage	education: high, low	low	$p > 0.05$	multivariate with inclusion > 60 years	

(continued next page)

Supplementary Table S2. (continued).

Authors, year, country	Study type	Sample in the analysis	Caries index	Effect measure	Education: categories of analysis Socioeconomic parameter (reference*)	Group with higher caries index	Bivariate analysis p value	Multivariate analysis p value	NOS
Eriksen et al. (1996), Portugal	Cross sectional	196	Decayed surfaces-DS	Mean (SD)	years at school: < 10 years, ≥ 10	<10 years	14.5 (12.7) p > 0.05	no test	6
Faragó et al. (2012), Hungria	Cross sectional	792	Decayed teeth-DT	Mean (SD), Odds ratio OR (95%CI)	highest education level of father: primary*, secondary, high/university or tertiary	primary	8.47 (4.03) p > 0.05	Tertiary OR= 0.57 p > 0.05	7
			DMFT	Mean (SD), Odds ratio OR (95%CI)		primary	11.16 (5.61) p < 0.05	Tertiary OR= 0.38 p < 0.05	
Geyer et al. (2010), Germany	Cross sectional	925	DMFT (≤ 21, > 21)	Odds ratio OR (95%CI)	years of schooling: 12-13*, 10, 8-9 years	8-9 years	3.75 (1.99, 7.05) p < 0.05	2.95 (1.52, 5.74) p < 0.05	7
Gilbert et al. (2001), USA	Cohort	726	DF root surface	Percentage	high school graduate: yes, no	no high school graduate	p < 0.05	multivariate model no including variable education	8
Hahn et al. (1999), Germany	Cohort baseline	298	DMFT - decayed root	Beta	education	less schooling	not available	Root caries and education: Beta= 0.0129 p > 0.05	7
Hansen (1977), Norway	Cross sectional	60 males	DMFT	Mean (SD)	education: ≤10, >10	> 10 years	25.6 (3.34) p > 0.05	not available	6
			DT	Mean (SD)	education: ≤10, >10	≤ 10 years	6.1 (3.21) p > 0.05	not available	
		57 females	DMFT	Mean (SD)	education: ≤10, >10	≤ 10 years	25.8 (2.68) p > 0.05	not available	
			DT	Mean (SD)	education: ≤10, >10	> 10 years	5.6 (2.98) p > 0.05	not available	

(continued next page)

Supplementary Table S2. (continued).

Authors, year, country	Study type	Sample in the analysis	Caries index	Effect measure	Education: categories of analysis Socioeconomic parameter (reference*)	Group with higher caries index	Bivariate analysis p value	Multivariate analysis p value	NOS
Hessari et al. (2007), Iran	Cross sectional	2,068	DMFT for males	Mean (SD)	education: illiterate, low, medium, high	illiterate	11.4 (7.0) $p < 0.05$	not available	7
			DT for males	Mean (SD)	education: illiterate, low, medium, high	low	2.8 (2.6) $p < 0.05$	not available	
		4,676	DMFT for females	Mean (SD)	education: illiterate, low, medium, high	illiterate	11.7 (7.1) $p < 0.05$	not available	
			DT for females	Mean (SD)	education: illiterate, low, medium, high	Illiterate and low	2.8 (2.7) and 2.8 (2.8) $p < 0.05$	not available	
Holst and Schuller (2011), Norway	Cohort (results over 33 year)	Birth-cohorts in age 35–44 years old in 1983 n=300	DMFS	Mean	education: quartile (shortest education, second shortest education, second longest education, longest education)	Lowest educational quartile	does not describe values $p < 0.05$	not available	6
			Decayed surfaces-DS	Mean	education: quartile (shortest education, second shortest education, second longest education, longest education)	Lowest educational quartile	does not describe values $p < 0.05$	not available	
		Birth-cohorts in age 35–44 years old in 2006 n=158	DMFS	Mean	education: quartile (shortest education, second shortest education, second longest education, longest education)	DMFS was not related to education	does not describe values $p > 0.05$	not available	

(continued next page)

Supplementary Table S2. (continued).

Authors, year, country	Study type	Sample in the analysis	Caries index	Effect measure	Education: categories of analysis Socioeconomic parameter (reference*)	Group with higher caries index	Bivariate analysis p value	Multivariate analysis p value	NOS
Holst and Schuller (2012), Norway	Cohort Life course	Birth-cohorts: 1929-1938, 1939-1948, 1959-1960 (23-24, 34-44 and 45-54 years old in 1983 and 2006)	DMFS, DS	Regression coefficient (SE)	length education: lowest*, second lowest quartile, second highest quartile, highest quartile	-	-	-	6
		23 to 24 years old in 1983 n=773	DMFS	Regression coefficient (SE)	length education: lowest*, second lowest quartile, second highest quartile, highest quartile	lowest	not available	highest quartile -13.36 (2.48) $p < 0.05$	
			DS	Regression coefficient (SE)	length education: lowest*, second lowest quartile, second highest quartile, highest quartile	lowest	not available	highest quartile -0.93 (0.67) $p > 0.05$	
		35 to 44 years old in 1983 n=773	DMFS	Regression coefficient (SE)	length education: lowest*, second lowest quartile, second highest quartile, highest quartile	lowest	not available	highest quartile -11.98 (2.26) $p < 0.05$	
			DS	Regression coefficient (SE)	length education: lowest*, second lowest quartile, second highest quartile, highest quartile	lowest	not available	highest quartile -1.55 (0.64) $p < 0.05$	
		45 to 54 years old in 1983 n=675	DMFS	Regression coefficient (SE)	length education: lowest*, second lowest quartile, second highest quartile, highest quartile	lowest	not available	highest quartile -13.36 (2.48) $p < 0.05$	
			DS	Regression coefficient (SE)	length education: lowest*, second lowest quartile, second highest quartile, highest quartile	lowest	not available	highest quartile -0.93 (0.67) $p > 0.05$	
		In 2006, sample from the 1959-1960 (46-47 year-old) n=96 Combined datafile 1983 and 2006.	DMFS	Regression coefficient (SE)	length education: lowest*, second lowest quartile, second highest quartile, highest quartile	lowest	not available	highest quartile -11.93 (2.84) $p < 0.05$	
			DS	Regression coefficient (SE)	length education: lowest*, second lowest quartile, second highest quartile, highest quartile	lowest	not available	highest quartile -2.08 (0.42) $p < 0.05$	

(continued next page)

Supplementary Table S2. (continued)

Authors, year, country	Study type	Sample in the analysis	Caries index	Effect measure	Education: categories of analysis Socioeconomic parameter (reference*)	Group with higher caries index	Bivariate analysis p value	Multivariate analysis p value	NOS
Julihn et al. (2006), Sweden	Cross sectional	696	DMFS (< 10, ≥ 10)	Percentage, Beta coefficient	education level of father: ≤ 9 years, 10–12 years, > 12 years	≤ 9 years	p < 0.05	does not describe values p > 0.05	7
Lin et al. (2001), China	Cross sectional	1,573	DMFT	Mean (SD)	education: no schooling/primary, secondary, post-secondary	no schooling/primary	5.4 (0.2) p < 0.01	not available	7
Mamai-Homata et al. (2012), Grécia	Cross sectional	1,184	DMFT	Mean (SD)	educacion: ≤ 6 years*, 9 years, 12 years, > 12 years	≤ 6 years	15.51 (6.89) p > 0.05	not available	7
			Decayed teeth–DT	Mean	educacion: ≤ 6 years*, 9 years, 12 years, > 12 years	≤ 6 years	2.68 p > 0.05	not available	
			DMFS	Mean (SD), Odds ratio OR (95%CI)	educacion: ≤ 6 years*, 9 years, 12 years, > 12 years	≤ 6 years	55.86 (32.53) p < 0.001	> 12 years OR= 0.321 (0.193, 0.535) p < 0.001	
			Decayed surfaces-DS	Mean	educacion: ≤ 6 years*, 9 years, 12 years, > 12 years	≤ 6 years	6.27 p > 0.05	not available	
			RDFS	Mean (SD), Odds ratio OR (95%CI)	educacion: ≤ 6 years*, 9 years, 12 years, > 12 years	≤ 6 years (bivariate); ≤ 12 years (multivariate)	0.63 (2.02) p < 0.01	> 12 years OR= 0.346 (0.180, 0.664) p < 0.01	
			RDS	Mean	educacion: ≤ 6 years*, 9 years, 12 years, > 12 years	≤6 years	0.56 p > 0.05	not available	
Paulander et al. (2003), Sweden	Cross sectional	35 years n=142	DS	Mean (95%CI)	education: low, high	low	1.3 (-0.16, 2.69) p > 0.05	not available	6
		50 years n=406	DS	Mean (95%CI)	education: low, high	low and high	0.4 (0.17, 0.59) 0.4 (0.15, 0.70) p > 0.05	not available	

(continued next page)

Supplementary Table S2. (continued)

**Authors, year, country**	**Study type**	**Sample in the analysis**	**Caries index**	**Effect measure**	**Education: categories of analysis Socioeconomic parameter (reference*)**	**Group with higher caries index**	**Bivariate analysis p value**	**Multivariate analysis p value**	**NOS**
Quintero et al. (2014), Chile	Cross sectional	451	DMFT	Mean (SD), Beta (95%CI)	education: no formal studies, primary, secondary, higher	primary	15.51 (6.73) p > 0.05	Beta= -0.072 (-0.875, 0.731) p > 0.05	7
			Decayed teeth- DT	Mean (SD)	education: no formal studies, primary, secondary, higher	no formal	3.75 (3.50) p > 0.05	not available	
Roberts-Thomson and Stewart (2008), Australia	Cross sectional	644	DMFS	Mean (SD)	tertiary education: yes, no	no tertiary education	6.19 (7.79) p < 0.05	not available	7
			Decayed surfaces- DS	Mean (SD)	tertiary education: yes, no	no tertiary education	1.06 (2.55) p > 0.05	not available	
Schuller et al. (1999), Norway	Cross sectional	Analysis per year/n: 1983/792	DFS	Mean (95%CI)	education: low (≤12), high (≥13)	low	40.7 (39.1, 42.3) p < 0.05	not available	6
		1994/427	DFS	Mean (95%CI)	education: low (≤12), high (≥13)	low	22.3 (19.9, 24.7) p < 0.05	multivariate model no including education	
Senna et al. (2005), Italy	Cross sectional	Category military/n: call-up soldiers/867	DMFT	Mean (SD)	education : primary, secondary, high, degree	degree	5.17 (3.03) p < 0.05	not available	5
			DT	Mean (SD)	education : primary, secondary, high, degree	primary	1.89 (1.92) p < 0.05	not available	
		cadets/2,043	DMFT	Mean (SD)	education : primary, secondary, high, degree	degree	4.42 (3.03) p < 0.05	not available	
			DT	Mean (SD)	education : primary, secondary, high, degree	primary	0.57 (0.98) p > 0.05	not available	

(continued next page)

Supplementary Table S2. (continued).

Authors, year, country	Study type	Sample in the analysis	Caries index	Effect measure	Education: categories of analysis Socioeconomic parameter (reference*)	Group with higher caries index	Bivariate analysis p value	Multivariate analysis p value	NOS
Sgan-Cohen et al. (1999), Israel	Cross sectional	1,084	DMFT	Mean (SD)	years at school: < 12, 12, > 12	>12 years	not available	12.08 (6.1) p > 0.05	6
			Decayed teeth-DT	Correlation coefficient R, Mean (SD)	years at school: < 12, 12, > 12	negative correlation	R = -0.16 p < 0.001	mean=1.75 (2.4) adjusted for age and gender p < 0.001	
Sgan-Cohen et al. (2000), Israel	Cross sectional	7,132	DMFT	Mean (SD), Poisson regression	years of schooling: < 12, ≥ 12	≥ 12	8.54 (4.93) p < 0.05	does not describe values - p < 0.01	5
			DT		years at school: < 12, 12, > 12	< 12	3.47 (3.70) p < 0.05	does not describe values - p= 0.0001	
Skudutyte, Aleksejuniene and Eriksen (2000), Lithuania	Cross sectional	380	DMFT	Median	education: low (< 12 years), medium (12 to 14 years), high (>14 years)	high	19.0 p > 0.05	not available	6
			DT	Median	education: low (< 12 years), medium (12 to 14 years), high (> 14 years)	low	3 p<0.001	not available	
Skudutyte-Rysstad et al. (2009), Norway	Cross sectional	149	Decayed surfaces-DS (≥ 2, < 2)	Percentage, Odds ratio OR (95%CI)	education university: no*, yes	no university	p < 0.05	yes OR= 0.8 (0.3, 2.1) p > 0.05	6
Tervonen et al. (1991), Finland	Cross sectional	883	DT (< 7, ≥ 7)	Odds ratio OR (95%CI)	years of education	less education	not available	high education OR= 0.89 (0.83, 0.97) p < 0.05	7
Unel et al. (1999), Sweden	Cross sectional	919	DFT	Regression coefficient Beta= b	education: college, high/grammar, secondary, primary*	primary	not available	college - b= -6.2 p < 0.05	7
		513	DT	Regression coefficient b	education: college, high/grammar, secondary, primary*	primary	not available	college b= -4.4 p < 0.05	

(continued next page)

Supplementary Table S2. (continued).

**Authors, year, country**	**Study type**	**Sample in the analysis**	**Caries index**	**Effect measure**	**Education: categories of analysis Socioeconomic parameter (reference*)**	**Group with higher caries index**	**Bivariate analysis p value**	**Multivariate analysis p value**	**NOS**
Vano et al. (2014), Italy	Cross sectional	350	DMFT	Mean (SD)	education: elementary, middle, high, university	elementary	4.65 (2.42)<br>$p > 0.05$	not available	6
			DT	Mean (SD)	education: elementary, middle, high, university	elementary	1.98 (1.86)<br>$p > 0.05$	not available	
Varenne et al. (2006), Burkina Faso	Cross sectional	493	DMFT	Beta - linear regression	education: high, moderate, low*	high	not available	high education<br>Beta= 2.85<br>$p < 0.05$	7
			DT<br>(0, ≥ 1)	Odds ratio – OR	education: high, moderate, low*	high	not available	high education<br>OR= 2.99<br>$p < 0.05$	
Zini et al. (2012)a, Israel	Cross sectional	254	DMFT	Mean (95%CI), Beta (95%CI)	education: low*, academic, yeshiva	low	12.33 (11.35, 13.31)<br>$p < 0.001$	yeshiva<br>Beta= -2.70 (-4.72,-0.69)<br>$p < 0.05$	7
			DT	Mean (95%CI), Beta (95%CI)	education: low*, academic, yeshiva	low	1.35 (1.07, 1.63)<br>$p < 0.001$	yeshiva<br>Beta= -0.49 (-0.90, 0.01)<br>$p < 0.05$	
Zini et al. (2012)b, Israel	Cross sectional	248	DMFT<br>(<11, ≥11)	OR (95%CI)	education: low*, academic, high yeshiva	low	high yeshiva<br>OR= 0.31(0.14, 0.68)<br>$p < 0.05$	no test	7
Zini et al. (2013), Israel	Cross sectional	254	DMFT	Mean (95%CI), OR (95%CI)	education: high (academic and yeshiva), low*	low	12.33 (11.4, 13.3)<br>$p < 0.01$	high yeshiva<br>OR= 0.53 (0.30, 0.95)<br>$p < 0.05$	7
			DT	Mean (95%CI), OR (95%CI)	education: high (academic and yeshiva), low*	low	1.35 (1.1, 1.6)<br>$p < 0.01$	high yeshiva<br>OR= 0.54 (0.28, 1.05)<br>$p > 0.05$	

Risk of bias was assessed using the Newcastle-Ottawa (NOS) for observational studies (Wells et al. 2009) with scores for summarizing the multitude data. DMFT decayed missing filled teeth; DT decayed teeth; DS/DFS decayed (filled) surfaces; DMFS decayed missing filled surfaces; RDFS, RDS decayed (filled) surfaces root; DF decayed root; Mean (SD) standard deviation; (SE) standard error; RR risk ratio; 95%CI confidence interval; OR odds ratio; PR prevalence rate; R correlation coefficient.

Supplementary Table S3. Study characteristics and results reported of the occupational status.

Authors, year, country	Study type	Sample in the analysis	Caries index	Effect measure	Occupational status: categories of analysis Socioeconomic parameter (reference*)	Group with higher caries index	Bivariate analysis p value	Multivariate analysis p value	NOS
Brennan et al. (2007), Australia	Cross sectional	709	DMFT	Mean (SE), Beta coefficient	concession card holder: yes, no* (card holder for disadvantged group)	yes	17.53 (0.54) $p < 0.05$	not available	7
			Decayed teeth-DT	Mean (SE), Beta coefficient	concession card holder: yes, no* (card holder for disadvantged group)	yes	0.72 (0.13) $p < 0.01$	yes Beta= 0.33 $p > 0.05$	
Broadbent et al. (2016), New Zealand	Cohort Life course	878	Decayed surfaces-DS	Beta coefficient (95%CI)	occupation during the age 26 and 32 years and oral health outcomes the age 38 years	lowest occupational status	not available	Beta= -1.186 (-2.143, -0.367) $p < 0.05$	8
Hescot et al. (1997), France	Cross sectional	1,000	DMFT	Mean (SD)	occupational group: high, medium, low	low	14.8 (6.2) $p < 0.05$	not available	6
			DT	Mean (SD)	occupational group: high, medium, low	low	1.3 (2.0) $p > 0.05$	not available	
Julihn et al. (2006), Sweden	Cross sectional	696	DMFS (<10, ≥10)	Percentage Beta coefficient	occupational status of mother: unemployed, laborer, white-collar worker	white-collar	$p < 0.05$	does not describe values $p > 0.05$	7
			DMFS (< 10, ≥ 10)	Percentage Beta coefficient	occupational status of father: unemployed, laborer, white-collar worker	laborer	$p < 0.01$	does not describe values $p > 0.05$	
Quintero et al. (2014), Chile	Cross sectional	450	DMFT	Mean (SD)	employed, self-employed, housewife, retired, other	retired	21.50 (5.91) $p < 0.05$	not available	7
			DT	Mean (SD)	employed, self-employed, housewife, retired, other	retired	3.25 (2.75) $p > 0.05$	not available	

(continued next page)

Supplementary Table S3. (continued).

**Authors, year, country**	**Study type**	**Sample in the analysis**	**Caries index**	**Effect measure**	**Occupational status: categories of analysis Socioeconomic parameter (reference*)**	**Group with higher caries index**	**Bivariate analysis p value**	**Multivariate analysis p value**	**NOS**
Roberts-Thomson and Stewart (2008), Australia	Cross sectional	644	DMFS	Mean (SD) Beta (SE)	employed: yes, no*	no	6.94 (9.72) p < 0.05	Yes Beta= -0.05 (1.04) p > 0.05	7
			Decayed surfaces-DS	Mean (SD) Beta (SE)	employed: yes, no*	no	1.16 (5.83) p < 0.05	Yes Beta= 0.09 (0.34) p < 0.05	
			DMFS	Mean (SD)	gov't benefits: yes, no*	yes	8.77 (12.55) p < 0.05	Yes Beta= 0.15 (0.95) p < 0.01	
			DS	Mean (SD)	gov't benefits: yes, no*	yes	1.68 (4.94) p < 0.05	Yes Beta= 0.04 (0.31) p > 0.05	
Unel et al. (1999), Sweden	Cross sectional	919	DFT	Regression coefficient Beta	occupational status: white-collar works in leading positions, white-collar workers, entrepreneurs, blue-collar workers*	blue-collar workers	not available	entrepreneurs Beta= -1.3, p > 0.05	7
		513 (DT=0 removed)	Decayed teeth-DT	Regression coefficient Beta	occupational status: white-collar works in leading positions, white-collar workers, entrepreneurs, blue-collar workers*	blue-collar workers	not available	entrepreneurs Beta= -5.7 p < 0.05	
Varenne et al. (2006), Burkina Faso	Cross sectional	493	DMFT	Regression coefficient - Beta	occupation: shopkeeper, government employee, smallholder/craftsman, housewife, farmer/breeder*	farmer/breeder	not available	shopkeeper Beta= -1.19 p > 0.05	7
			DT (= 0, ≥ 1)	Odds ratio OR (95%CI)	occupation: shopkeeper, government employee, smallholder/craftsman, housewife, farmer/breeder*	government employee	not available	shopkeeper OR= 0.75, p > 0.05 government employee OR= 5.26, p < 0.01	
Zini et al. (2012)b, Israel	Cross sectional	248	DMFT <11,≥ 11	Median	employment status: employed* unemployed	unemployed	OR= 0.66 (0.37, 1.18), p > 0.05	not available	7

Risk of bias was assessed using the Newcastle-Ottawa (NOS) for observacional studies (Wells et al. 2009) with scores for summarizing the multitude data. DMFT decayed missing filled teeth; DT decayed teeth; DS/DFS decayed (filled) surfaces; DMFS decayed missing filled surfaces; RDFS, RDS decayed (filled) surfaces root; DF decayed root; Mean (SD) standard deviation; (SE) standard error; RR risk ratio; 95%CI confidence interval; OR odds ratio; PR prevalence rate; R correlation coefficient.

Supplementary Table S4. Study characteristics and results reported of the socioeconomic status.

Authors, year, country	Study type	Sample in the analysis	Caries index	Effect measure	Socioeconomic status: categories of analysis Socioeconomic parameter (reference*)	Group with higher caries index	Bivariate analysis p value	Multivariate analysis p value	NOS
Berset et al. (1996), Norway	Cross sectional	120	Decayed surfaces-DS	Mean (SD)	social class: low, medium, high	low	low 3.4 (4.1) $p < 0.001$	does not describe values multiple regression	7
Bille (1980), Denmark	Cohort	313	DMFS	Mean	parent's socioeconomic status: high, medium, low, unknown	low	18 $p > 0.05$	not available	6
			DMFS	Mean	subject's own socioeconomic status: high, medium, low, unknown	low	21 $p < 0.01$	not available	
Bjertness et al. (1992), Norway	Cohort	81	Decayed teet-DT	Mean (SD)	social class: class 1, class 2, class 3	class 1	1.46 (0.52) $p > 0.05$	not available	8
Chandra Shekar and Reddy (2011), India	Cross sectional	1,187	DMFT	Mean (SD)	socioeconomic status (SES): upper, upper middle, lower middle, upper lower, lower	lower middle	2.51 (3.23) $p > 0.05$	not available	6
			Decayed teeth-DT	Mean (SD)	socioeconomic status (SES): upper, upper middle, lower middle, upper lower, lower	upper lower	1.05 (1.66) $p < 0.001$	not available	
Doughan et al. (2000), Lebanon	Cross sectional	401	DMFT	Mean (SD)	socio-economic status: low, middle, high (index was formulated, based on the occupation and education of the subject)	low	17.4 (7.4) $p < 0.05$	not available	7
			Decayed teeth-DT	Mean (SD)	socio-economic status: low, middle, high (index was formulated, based on the occupation and education of the subject)	low	5.7 (5.7) $p < 0.05$	not available	

(continued next page)

Supplementary Table S4. (continued).

**Authors, year, country**	**Study type**	**Sample in the analysis**	**Caries index**	**Effect measure**	**Socioeconomic status: categories of analysis Socioeconomic parameter (reference*)**	**Group with higher caries index**	**Bivariate analysis p value**	**Multivariate analysis p value**	**NOS**
Eriksen et al. (1996), Portugal	Cross sectional	196	Decayed surfaces- DS	Mean (SD), beta	social class: class 1, class 2, class 3	class 3 (high)	15.7 (13.2) $p > 0.05$	Beta= 0.08 $p > 0.05$	6
Geyer *et al.* (2010), Germany	Cross sectional	925	DMFT (≤21, >21)	Odds ratio OR (95%CI)	cumulative effects: income + educational level 1 = highest socioeconomic positions*, intermediate positions, lowest positions	lowest positions	6.06 (2.06, 17.87)	not given non significant	7
Marcenes and Sheiham (1992), Brazil	Cross sectional	164	DMFS	Correlation coefficient- R, Beta (SE)	socio-economic status by ABA-ABIPEME (resources and educational level)	lowest socio-economic status	R= -0.19 $p < 0.05$ Beta= -0.36 (0.14) $p < 0.05$	Beta= -0.31 (0.17) $p < 0.05$	7
Meyer et al. (1983), Portugal	Cross sectional	73	DMFT	Mean (SD) and range	lower socioeconomic status (manual laborers) and higher socioeconomic status (the first three classes of students graduating from the new dental school in Lisbon)	students	15.9 (6.3) and 2-28 $p < 0.01$	not available	6
			DMFS	Mean (SD) and range	lower socioeconomic status (manual laborers) and higher socioeconomic status (the first three classes of students graduating from the new dental school in Lisbon)	students	42.6 (25.0) and 3-103 $p < 0.01$	not available	
Shearer et al. (2011), New Zealand	Cohort	932	DMFS	Rate ratio RR (95%CI)	socioeconomic (SES) at phase 32 year: low, medium, high*	low	not available	1.15 (0.95, 1.40)	8
Thomson et al. (2004), New Zealand	Cohort	789	Decayed surfaces- DS	Mean	status socioeconomic - SES group at age 5 years: high, low	low	not available	1.88 $p < 0.05$	9
			Decayed surfaces- DS	Mean	SES trajectory (early childhood SES to age-26-year SES): high-high, low-high, high-low, low-low	low-low	not available	2.05 $p < 0.001$	

Risk of bias was assessed using the Newcastle-Ottawa (NOS) for observacional studies (Wells et al. 2009) with scores for summarizing the multitude data. DMFT decayed missing filled teeth; DT decayed teeth; DS/DFS decayed (filled) surfaces; DMFS decayed missing filled surfaces; RDFS, RDS decayed (filled) surfaces root; DF decayed root; Mean (SD) standard deviation; (SE) standard error; RR risk ratio; 95%CI confidence interval; OR odds ratio; PR prevalence rate; R correlation coefficient.

Supplementary Table S5. Study characteristics and results reported of the collective indicators and other.

Authors, year, country	Study type	Sample in the analysis	Caries index	Effect measure	Collective indicators	Group with higher caries index	Bivariate analysis p value	Multivariate analysis p value	NOS
Bernabe et al. (2009) , 18 countries	Ecological	18 countries	DMFT	Correlation coefficient-R	Gross domestic product - GDP per capita; Gross national income - GNI per capita in 2000 (in dollars); Gini coefficient	> GDP<br>> GNI<br>< Gini	GDP R= 0.09 p > 0.05<br>GNI R= 0.10 p > 0.05<br>Gini R= -0.66 p < 0.01	not available	5
			Decayed teeth - DT	Correlation coefficient-R	Gross domestic product - GDP per capita; Gross national income - GNI per capita in 2000 (in dollars); Gini coefficient	< GDP<br>< GNI<br>> Gini	GDP R= -0.20 p > 0.05<br>GNI R= -0.31 p > 0.05<br>Gini R= 0.18 p > 0.05	not available	
Celeste et al. (2009), Brazil	Cross sectional	20,194	Decayed teeth - DT	Odds ratio OR (95%CI) Multilevel analysis	Gini and individual income	> Gini and < income	not available	1.16 (1.06, 1.26) for each 10 points increase in Gini scale	5
			Decayed teeth - DT	OR (95%CI) Multilevel analysis	synergy index = Gini (< 0.56, > 0.56) and individual income > 850, < 850	individual income < 850 and Gini > 0.56	not available	individual income < 850 and Gini > 0.56 3.03 (2.68, 3.43)	
Gao et al. (2013), China	Cross sectional	122 Women Foreign domestic worker	DMFT	Mean, Regression coefficient Beta (95%CI)	having own room in employer's home: yes, no	no	7.24 p<0.01	Yes Beta= -2.459 (-0.980, -3.938) p < 0.01	7

Risk of bias was assessed using the Newcastle-Ottawa (NOS) for observacional studies (Wells et al. 2009) with scores for summarizing the multitude data. DMFT decayed missing filled teeth; DT decayed teeth; 95%CI confidence interval; OR odds ratio; R correlation coefficient, GDP Gross domestic product; GNI Gross national income; Gini – index used to measure inequality of income distribution.
